# Relationship Between the Digital Assessment of Cognition and Paper and Pencil Neuropsychological Tests

**DOI:** 10.1002/alz70857_104820

**Published:** 2025-12-25

**Authors:** Ileana De Anda‐Duran, Phillip H Hwang, Laura A Salciunas, Aeysha J Brown, Elizabeth Leverant, Mitchel A. Kling, Rodney Swenson, David J. Libon

**Affiliations:** ^1^ Celia Scott Weatherhead Tulane University School of Public Health and Tropical Medicine, New Orleans, LA, USA; ^2^ Department of Epidemiology, Boston University School of Public Health, Boston, MA, USA; ^3^ New Jersey Institute for Successful Aging, Rowan University, Stratford, NJ, USA; ^4^ Rowan‐Virtua School of Osteopathic Medicine, New Jersey Institute for Successful Aging, Stratford, NJ, USA; ^5^ Department of Psychiatry, University of North Dakota Medical School, Fargo, ND, USA

## Abstract

**Background:**

The Digital Assessment of Cognition (DAC) is a self‐administered digital neuropsychological protocol requiring approximately 7 minutes to administer, either in‐clinic or remotely. The DAC yields numerous metrics including episodic memory and working memory indices. The current research assessed relationships between the DAC and traditional paper/pencil neuropsychological tests.

**Method:**

171 memory clinic patients were assessed with the DAC. A k‐mean cluster analysis classified patients presenting with dysexecutive MCI (*n* = 34); amnestic MCI (*n* = 20); mixed MCI (*n* = 43); dementia (*n* = 26,) and normal cognition (NC, *n* = 48). A portion of these patients (*n* = 107) were assessed with a protocol of paper/pencil neuropsychological tests assessing five neurocognitive domains: attention (WAIS‐IV Digits Forward, Trails A); working memory (WMS‐IV Symbol Span, letter fluency), processing speed (Trails B, WAIS‐III Digit Symbol), language (Boston Naming Test, WAIS‐III Similarities), and episodic memory (CVLT‐9 delayed free recall/recognition). Using normative values, tests were averaged to create five paper/pencil indices.

**Result:**

The concordance between k‐mean and paper/pencil classification was 89%. When a linear, stepwise regression analysis (DAC memory index = dependent variable; five paper/pencil indices = independent variables) was conducted, the paper/pencil episodic memory index entered first (beta=0.538, *p* <0.001) followed by the paper/pencil language index (beta=0.302, *p* <0.001). When a similar regression analysis for the DAC executive index was conducted, the paper/pencil working memory index entered first (beta=0.503, *p* <0.001), followed by the paper/pencil attention index (beta=0.288, *p* <0.001), followed by the paper/pencil memory index (beta=0.163, *p* <0.017). A multinomial logistic regression analysis (NC = reference group) found that the paper/pencil attention (Wald=6.15, *p* <0.013) and working memory indices (Wald=7.88, *p* <0.005) classified patients into the dMCI group; only the paper/pencil episodic memory index (Wald=15.08, *p* <0.001) classified patients into the aMCI group; both the paper/pencil working memory (Wald=11.02, *p* <0.001) and episodic memory indices (Wald=5.43, *p* <0.020) classified patients into the mixed MCI group; and the paper/pencil working memory (Wald=9.11, *p* <0.001), language (Wald=3.72, *p* <0.050) and episodic memory indices (Wald=9.49, *p* <0.001) classified patients into the dementia group

**Conclusion:**

These data provide evidence for both the construct and criterion validity of the DAC. The parsimony and ease associated with DAC administration/scoring suggests that the DAC can be deployed as a frontline assessment to flag emergent cognitive impairment.

